# Sonodynamic therapy with a single neoadjuvant, diffuse delivery of low-intensity ultrasound with 5-ALA in treatment naïve glioblastoma results in tumor-specific cytotoxic edema and increased apoptosis

**DOI:** 10.1007/s11060-025-04957-7

**Published:** 2025-02-04

**Authors:** Walter Stummer, Mirjam Gerwing, Sabriye Sennur Bilgin, Christian Thomas, Javier Villanueva-Meyer, Vijay Agarwal, Louise Stögbauer, Juliane Schroeteler, Michael Müther

**Affiliations:** 1 Department of Neurosurgery, University Hospital Münster, Albert-Schweitzer-Campus 1, 48149 Münster, Germany; 2 Department of Radiology, University Hospital Münster, Münster, Germany; 3 Institute of Neuropathology, University Münster, Münster, Germany; 4 Department of Radiology, University of California San Francisco, San Francisco, CA USA; 5 Montefiore Health Center, Department of Neurological Surgery, New York, NY USA; 6 Department of Neurosurgery, Klinikum Osnabrück, Osnabrück, Germany

**Keywords:** Glioblastoma, Aminolevulinic acid, Neoadjuvant therapies, Caspases, Cerebral blood volume, Low-intensity non-targeted ultrasound

## Abstract

**Purpose:**

Sonodynamic therapy, which combines a tumor cell-selective sonosensitizer with ultrasound, is gaining attention as a promising new treatment approach for glioblastoma. The objective of this case study is to report on the first applications of 5-aminolevulinic acid (5-ALA) in combination with low-intensity, non-targeted ultrasound as neo-adjuvant treatment in therapy naïve glioblastoma.

**Methods:**

Three patients with therapy naïve newly diagnosed glioblastoma were treated once before cytoreductive surgery with 5-ALA in combination with hemispheric, low-intensity, non-targeted ultrasound, assuming cell death to be triggered by non-ablative activation of 5-ALA-induced, tumor selective porphyrins.

**Results:**

No adverse effects were noted. Post-procedural MRI indicated a decrease in apparent diffusion coefficient values in tumors, suggesting cytotoxic effects. Relative cerebral blood volumes and leakage were increased for two patients with available perfusion imaging. Tissue obtained during surgery suggested increased cleaved-caspase III expression, a marker of apoptosis.

**Conclusion:**

We saw an immediate marked imaging response indicating cytotoxic edema and indications of a histopathology response from just a single treatment. Correlation to clinical outcomes and extension of overall survival remains to be seen. A Phase 1 safety study has been submitted for regulatory approval.

**Supplementary Information:**

The online version contains supplementary material available at 10.1007/s11060-025-04957-7.

## Background

High-grade gliomas are the most common primary brain tumors in adults, the most malignant type being glioblastoma [1]. With maximal safe resection and combined radio- and chemotherapy, median overall survival for newly diagnosed glioblastoma is less than 24 months [1, 2]. The extent of resection is closely related to survival, however, for functional reasons and associated morbidity the entirety of the enhancing portion of the tumor can only be resected in approximately 50% of patients [3]. After primary therapy, there is no clear consensus on the standard of care for recurrent disease, and randomized controlled trials for recurrent glioblastoma have consistently failed, rendering recurrent glioblastoma rapidly lethal [4, 5]. 

Five-aminolevulinic acid (5-ALA) is an endogenous amino acid precursor to heme and, when administered in excess, is selectively metabolized via heme metabolism to porphyrins, particularly protoporphyrin IX (PpIX), within malignant glioma cells. Accumulation elicits violet-red fluorescence when illuminated with blue light (405 nm) [6] which, when used for fluorescence-guided resections (FGR), allows for an increase in the extent of resection of the tumor [7, 8]. Both the United States Food and Drug Administration (FDA) and the European Medicines Agency (EMA) have approved 5-ALA-FGR, which is now considered the standard of care at most academic institutions.

In addition to facilitating FGR, 5-ALA-induced porphyrins in conjunction with oxygen may be activated by specific wavelengths of light (635 nm) resulting in selective toxicity to glioma cells via the local generation of reactive oxygen species (ROS) [9]. Promising pre-clinical data are available and clinical trials are ongoing [10–12]. Notably, ultrasound also appears to activate ALA-induced porphyrins with ensuing ROS generation, inducing cell death primarily through apoptosis [13, 14]. To this end, sonodynamic therapy (SDT), i.e. the combination of a tumor cell selective sonosensitizer and non-ablative, diffuse, low-intensity ultrasound, is emerging as a novel treatment option for glioblastoma [14]. In contrast to photodynamic therapy, using application of circumscribed optical radiation, SDT is capable of treating larger, more diffuse target volumes located more deeply in the skull in a non-invasive manner. This approach may offer an optimal means of addressing the challenges presented by diffuse and large lesions.

## Methods

Three patients with imaging diagnosis of glioblastoma and anticipated subtotal removal due to their eloquent location (Table 1) were treated in a compassionate setting as legally acknowledged in Germany [15]. The aim of therapy was to pretreat non-resectable tumor areas to bridge the time to radio- and chemotherapy and thus improve prognosis. Written informed consent from each patient was obtained in accordance with the Declaration of Helsinki. The local ethical committee gave consent for publication of our observations (registration number 2024-056-f-S). The authors of this report vouch for the accuracy of the data and the fidelity of treatment.

### Treatment specifics and tissue Processing

Before the planned surgery, patients were treated with a single course of diffuse non-ablative low-intensity ultrasound via the CV01 device (Alpheus Medical, North Oakdale, MN, USA). Prior to this, 5-ALA sensitization at the approved dose fluorescence-guided resection (Gliolan^®^ 20 mg/kg body weight, Medac, Wedel, Germany) was administered p.o. 6 h before SDT. The patient’s hair was shaved at the intended treatment sites across the involved hemisphere. The ultrasound transducer was sequentially placed at 10 different treatment sites across the hemisphere in a pre-defined manner to allow for whole-hemisphere treatment. Total treatment time per site was 12 min. Ultrasound was delivered at an acoustic intensity of 2–10 watts (W)/square centimeter (cm²) with a frequency of 0.750–1.1 megahertz (MHz). Ultrasound delivery includes a low duty cycle pulsed regimen that targets Isppa of 10 W/cm2 (~ 550 kPa) and a mechanical index (MI) of approximately 0.6 in situ (in the diseased tissue). Skull thickness at each of the ten treatment sites is evaluated using pre-procedural CT imaging, and the ultrasound output is scaled according to skull thickness and expected transmission losses to target I_sppa_ of 10 W/cm2 in situ. Patients were closely followed for neurological sequelae for 24 h. 24–48 h post-SDT, all patients received brain MRI per clinical standard, including diffusion-weighted, and, in two cases, perfusion imaging [16]. Standard of care debulking surgery was performed 3 to 4 days after sonication, using FGR, neuronavigation, imaging ultrasound, and monitoring/mapping techniques. Tissue samples from the peripheral invasive tumor zone of each patient were obtained for neuropathological and immunohistochemical analysis. All patients consequently began standard adjuvant radio- and chemotherapy without delay (within 4 weeks).

### Imaging

Imaging was interpreted by an independent radiologist [MG]. Quantitative analysis of the apparent diffusion coefficient (ADC) maps was performed using ImageJ (Version 1.54i) and GraphPadPrism (Version 10.2.1, GraphPad Software Inc., San Diego, CA, USA), with the region of interest covering the entire gadolinium contrast enhancing and FLAIR hyperintense tumor excluding areas of necrosis [17]. Tumor perfusion was analyzed using a commercial platform (IntelliSpace Portal 12.1, Philips Healthcare) with the region of interest covering the entire gadolinium contrast-enhancing tumor. Relative cerebral blood volume (rCBV) was defined as the total volume of flowing blood in a given volume of the brain and was calculated by the ratio of two areas: (1) the area under the time attenuation curve of the tissue and (2) the area under the time attenuation curve of the reference vein normalized by the hematocrit value. Corrected rCBV values were the extracted values corrected for contrast agent leakage, representing the permeability of the vessels in the defined volume.

### Tissue analysis

Pathologic diagnosis was confirmed by an independent neuropathologist [CT] based on the 2021 WHO classification for CNS tumors. Immunohistochemical analysis was carried out in a blinded fashion using Halo v3.6.4134 Image Analysis artificial intelligence software (Indica Labs, Albuquerque, New Mexico). Positive cleaved caspase 3 staining (anti-cleaved-caspase 3, Product Number 9664, Cell Signaling Technology, Inc.) was identified using a Halo area quantification algorithm (v2.4.3) by first defining the color settings for the hematoxylin counterstain and then the brown diaminobenzidine (DAB). Cells were counted in 10 high-power fields (40x magnification) in separate biopsy specimens from each patient.

In one patient (patient 2) histopathology was available from a biopsy performed 7 days before SDT for the purpose of pathologic diagnosis, which was used as a control sample. For functional reasons, intra-individual control tissue was not obtained.

## Results

**Fig. 1:**
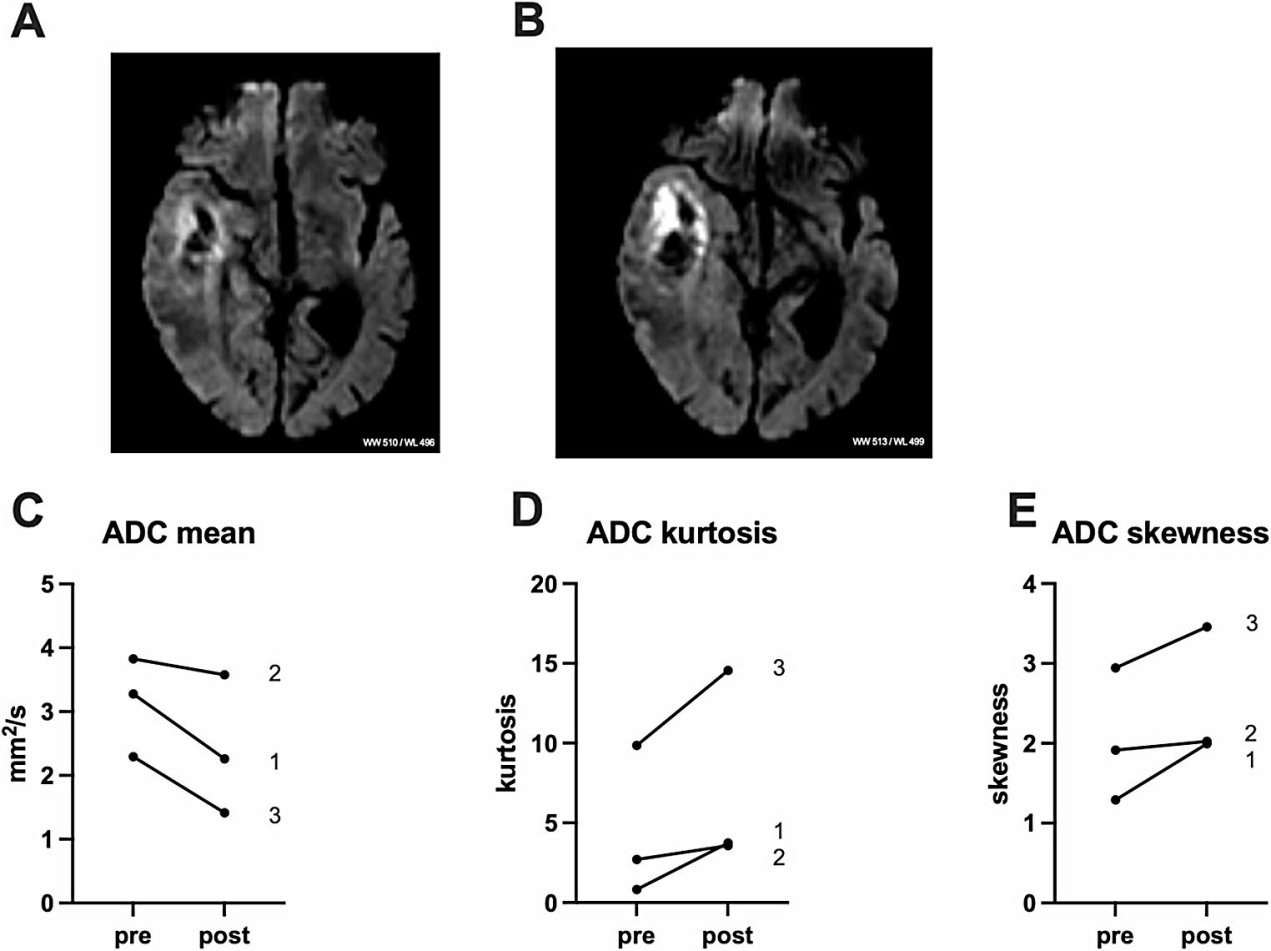
Illustrative example of diffusion imaging of patient 1 before (**A**) and after (**B**) sonication (TR: 2658.3 ms; TE: 80.8 ms, ST: 6 mm / SP: 7 M). Change in diffusion imaging values ADC mean, ADC kurtosis and ADC skewness in all patients, labeling refers to patient number (**C**-**E**, respectively). Paired t-testing resulted in p 0.09 (**C**), p 0.12 (**D**) and p 0.13 (**E**). ADC, apparent diffusion coefficient

**Fig. 2:**
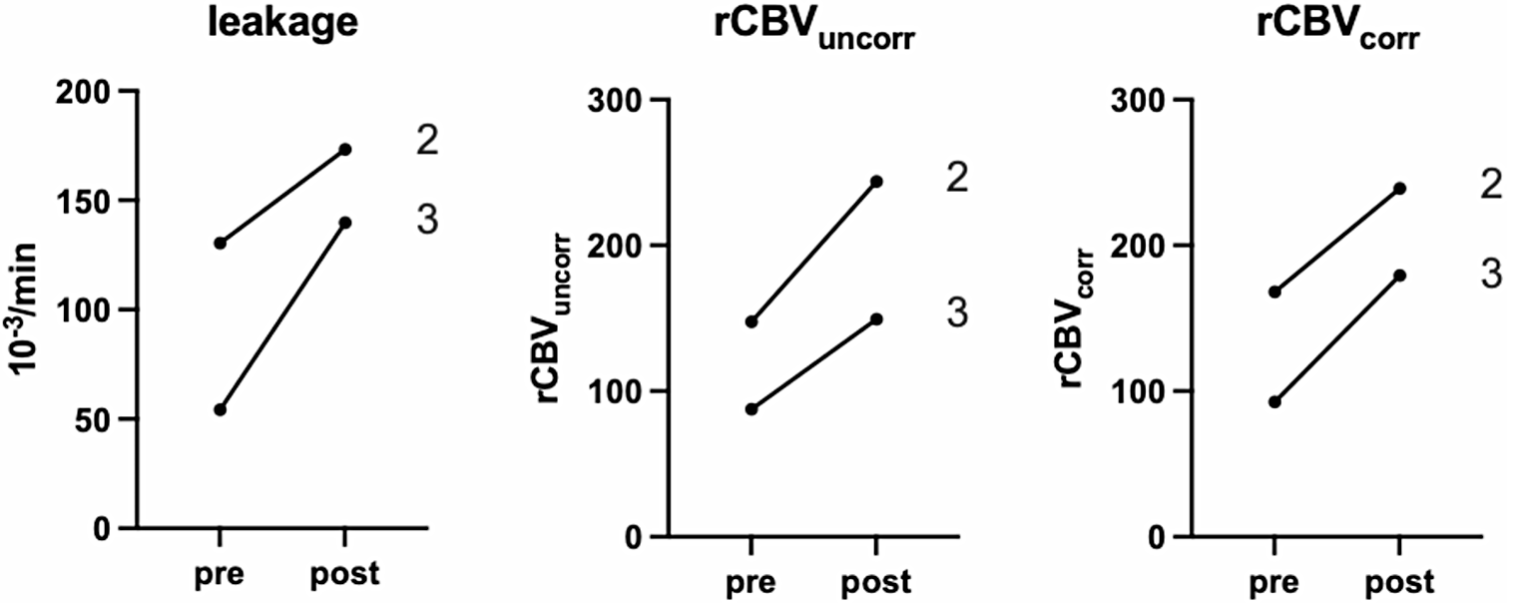
Change in tumor perfusion. Labeling refers to patient number. Paired t-testing resulted in p 0.12 (leakage), p 0.14 (rCBV_uncorr_) and p 0.06 (rCBV_corr_). rCBV_uncorr_, uncorrected relative cerebral blood volume; rCBV*corr*, corrected relative cerebral blood volume

**Fig. 3:**
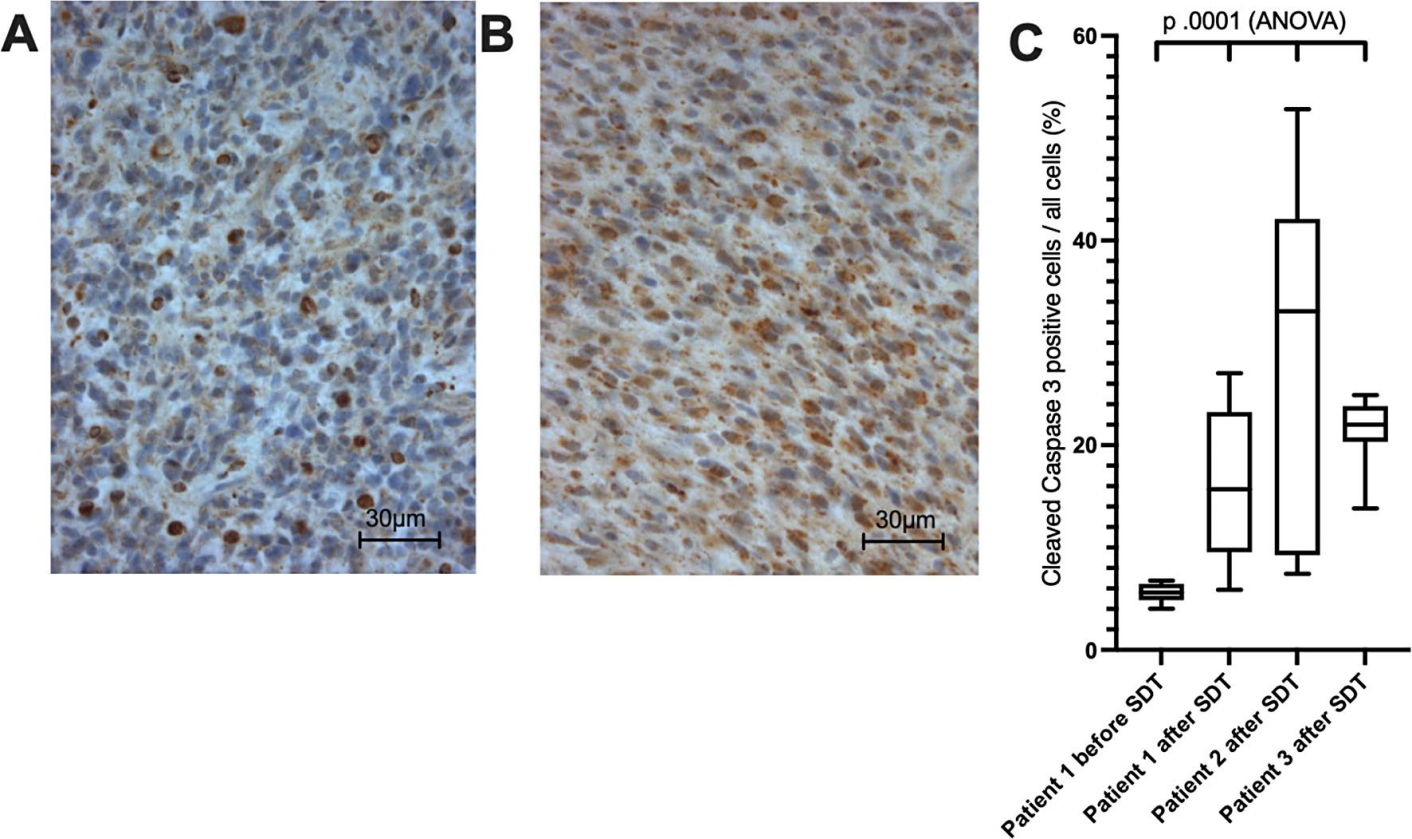
Anti Cleaved Caspase 3 immunohistochemistry in patient 1 before (**A**) and after (**B**) SDT. Comprehensive tissue analysis of all patients (**C**)

All three SDT applications (once per patient) were performed as planned without any observed adverse events. Early post- sonication MRI (less than 48 h) revealed no gross increase of edema or tumor mass (Supplement). Diffusion weighted imaging showed a distinct increase in signal and ADC demonstrated a corresponding decrease in signal confined to the tumor margins, which was consequently quantified (Fig. 1). In the patients with evaluable pre- and post-sonication perfusion studies (patients 2 and 3), we saw a measurable increase in leakage and perfusion in both tumors (Fig. 2). Normal brain did not show any noticeable alterations in anatomic imaging or on diffusion weighted or perfusion imaging between pre- and post-treatment MRI. Neuropathological evaluation confirmed glioblastoma in all cases. All cases post-SDT treatment demonstrated high cleaved caspase 3 levels. Figure 3 demonstrates cleaved caspase 3 expression, in one patient 7 days prior to sonication and after surgery, and in 2 patients after surgery. Table 1 summarizes latencies between interventions, imaging, and clinical data.

**Table 1:** Patient characteristics

Patient	Age (years)	Sex	Tumor location	Time between pre-sonication MRI and sonication (days)	Time between sonication and post- sonication MRI (days)	Time between sonication and surgery (days)	Resection status	Adjuvant therapy	Follow-up and status
1	83	male	Right temporal	1	2	3	subtotal	Radiochemotherapy with temozolomide (MGMT promotor not methylated)	lost to follow-up after RCT
2	62	female	Left parietal	1	4	5	subtotal	Radiochemotherapy with temozolomide and lomustine (MGMT promotor methylated)	Stable disease, second cycle of chemotherapy
3	56	male	Right temporal	1	2	3	subtotal	Radiochemotherapy with temozolomide (MGMT promotor not methylated)	Stable disease, first cycle of chemotherapy

## Discussion

We here report our initial observations of whole-hemisphere, non-ablative, low-intensity, diffuse ultrasound with 5-ALA in treatment naïve glioblastoma with invasive characteristics not allowing for gross total resection. We saw an immediate marked imaging response indicating cytotoxic edema and indications of a histopathology response from just a single treatment. No adverse events were noted.

Sonodynamic therapy using ultrasound and 5-ALA is presently being explored in both pre-clinical and clinical settings for brain tumors using both low-intensity focused ultrasound (LIFU) and magnetic resonance image-guided focused ultrasound (MRgFUS) together with 5-ALA (NCT06039709, NCT04845919). With both studies the ultrasound application is focused and requires MRI guidance [18]. CV01 (Alpheus Medical, North Oakdale, MN, USA), in contrast, delivers diffuse low-intensity ultrasound to cover the entire hemisphere, without the need for MRI, which makes intuitive sense considering the highly infiltrative nature of the disease [19]. Thus, the methodology can be referred to as low-intensity diffuse ultrasound (LIDU). Many even consider glioblastoma a “whole-hemisphere/brain disease”, as human glioma cells can be isolated distant from areas of enhancement or T2/FLAIR signals on MRI in histologically normal brain [20]. This underscores the need for a diffuse treatment for malignant gliomas, and a pivotal reason why focal treatments in the past have failed. A phase-1-study to evaluate 5-ALA combined with CV01 delivery of LIDU in recurrent high-grade glioma has just completed recruitment (NCT05362409).

While there is disparity reported for the direction of ADC changes (increased or decreased) in the context of post-radiotherapy imaging, lower ADC values have been shown to distinguish responders from non-responders at one week post-radiotherapy MRI [21, 22]. These reported findings underscore the importance of timing when measuring ADC change with ADC reductions detected as early as the hours following treatment and sustained for 3–5 days with a subsequent normalization to baseline and ultimate increase in the weeks to months following treatment. Similarly, reduced diffusion is commonly seen in the ablation field following laser interstitial thermal therapy [23]. Here, in the 24 h post-SDT MRI, the observed findings of reduced diffusion are in line with those seen in other ablative therapies.

Although this imaging was obtained in only two of the patients, it is worth noting that MRI perfusion metrics have been demonstrated to increase in the early (hours to days) post-treatment period following other ablative therapies (e.g. radiotherapy) with a subsequent decrease at the typical post-treatment imaging window after weeks to months, and are associated with response to therapy [24, 25]. 

SDT treatments in this study were conducted on a mobile ultrasound unit and do not require any headframes or anesthesia (as they are delivered in a “helmet-like” device), are conducted in just over two hours with the patient awake, and can be applied repeatedly. This is important, as due to the aggressive growth rate of malignant gliomas, repeat treatments are likely necessary for tumor control [26]. Owing to the ease of administration and the noiseless and painless nature of the treatment in an outpatient setting, chances of patient compliance and adherence are also increased, which has been a significant problem in other treatments.

In these three patients, we show that a single, transcranial, hemispheric treatment results in biological response, as determined both from imaging and as suggested by histopathology. On MRI, ADC values decreased with a corresponding increase in diffusion weighted imaging, indicating cytotoxic edema of cells related to the tumor, whereas perfusion and leakage in the region of the tumor were enhanced. The significance of the blood-brain barrier permeability increase is unknown. Increased perfusion and edema have been observed with focused ultrasound, which is being used for opening the blood-brain barrier, albeit using microbubbles [27]. Here we witness such phenomena in both patients where evaluable perfusion studies were available, signifying additional biological reactions from a single treatment with 5-ALA-SDT. Such changes after SDT could allow for better delivery of intravenous cytotoxic drugs or increase tumor oxygenation during radiotherapy [28]. Whether the imaging alterations indicate durable tumor cell cytotoxicity remains to be seen, however, the changes were rapid and drastic. Additionally, although the entire hemisphere was treated, the normal brain did not show any noticeable alterations in anatomic imaging or on diffusion weighted or perfusion imaging between pre- and post-treatment MRI. Regarding surgery in our three patients we also noted no appreciable differences to our experience of glioblastoma surgery without pre-operative SDT, but this needs to be assessed in the future.

Cleaved-Caspase 3 activation is related to apoptosis [29], and we observed increased levels after treatment in one patient where control tissue was available from a pre-treatment biopsy done 7 days before. In the other two patients, caspase-positive cell counts after a single SDT treatment were observed at a comparably elevated level. With such rapid and significant histopathologic changes correlating to the imaging, there could be the potential for a severe inflammatory or encephalopathic reaction as if seen with Car T cell therapies which might lead to patients’ deterioration [30]. No adverse events were observed however, indicating a lack of a bystander effect from the tumor cell death. This intuitively makes sense as the PPIX accumulation is in the mitochondria of the glioma, indicating that the generation of the reactive oxygen species and subsequent damage is likely occurring intracellularly with the tumor cells.

Importantly, the effects were seen with a single treatment only of 5-ALA in conjunction with CV01 delivered ultrasound. Early repeat administration of 5-ALA for FGR has not been observed to induce toxic adverse events [31]. In addition, cumulative toxicity induced by multiple sonications at non-therapeutic energy levels has not been described. One of the difficulties encountered previously with repeat treatments for malignant glioma is resistance, for instance with the use of temozolomide [32]. Acquired resistance to Temozolomide is often due to DNA repair systems, epigenetic modifications, and microRNAs [32]. As SDT relies on an enzymatic reaction as opposed to direct DNA modification, including a decreased expression of ferrochelatase in glioma cells, resistance is less likely [6]. 

This is a safety driven study looking for indicators of efficacy (ADC, perfusion changes, pathology). We envision adjuvant or neoadjuvant cyclic treatment in two to four weekly intervals. While we expect any viable tumor regions to maintain perfusion and thus oxygenation (our present results suggest that perfusion is actually enhanced) and we expect surviving tumor cells to accumulate PPIX. Since 5-ALA metabolism via the porphyrin pathway is always present in live cells, we do not expect any resistance mechanisms. However, this needs to be studied in ongoing investigations. In our in-vivo protocols (unpublished data, manuscript in preparation) on the combination of radiotherapy and 5-ALA we have noted no resistance.

Although the imaging and histopathologic responses reported in this study have been extraordinary, correlation to clinical outcomes and extension of overall survival remains to be seen. However, due to the rapid and significant nature of the data it warrants further evaluation and a Phase 1 safety study with neoadjuvant SDT in newly diagnosed malignant glioma has been submitted for regulatory approval.

## Electronic supplementary material

Below is the link to the electronic supplementary material.

Supplementary Material 1

## Data Availability

No datasets were generated or analysed during the current study.
