# Supplementary material for: Sonodynamic therapy with a single neoadjuvant, diffuse delivery of low-intensity ultrasound with 5-ALA in treatment naïve glioblastoma results in tumor-specific cytotoxic edema and increased apoptosis
Source: J Neurooncol. 2025 Feb 4;172(3):687–93. doi: 10.1007/s11060-025-04957-7 (PMC11968568; doi:10.1007/s11060-025-04957-7)
Supplement: Supplementary file 1 — Supplementary Material 1 [file 11060_2025_4957_MOESM1_ESM.pdf]

Supplement

Patient Number	FLAIR hyperintense volume before insonation (cc)	FLAIR hyperintense volume after insonation (cc)
1	260	151
2	394	391
3	68	56

Supplementary Table 1 FLAIR hyperintense perilesional volumes before and after insonation
